# Nephrotic Syndrome in Children: From Bench to Treatment

**DOI:** 10.4061/2011/372304

**Published:** 2011-08-28

**Authors:** J.-C. Davin, N. W. Rutjes

**Affiliations:** ^1^Academic Children's Hospital Reine Fabiola, Free University of Brussels, Av. Jean-Joseph Crocq 15, 1020 Brussels, Belgium; ^2^Department of Pediatric Nephrology, Emma Children's Hospital/Academic Medical Centre, Meibergdreef 9, 1105 AZ Amsterdam, The Netherlands; ^3^Department of Pediatric Nephrology, Emma Children's Hospital/Academic Medical Center, University of Amsterdam, The Netherlands

## Abstract

Idiopathic nephrotic syndrome (INS) is the most frequent form of NS in children. INS is defined by the association of the clinical features of NS with renal biopsy findings of minimal changes, focal segmental glomerulosclerosis (FSGS), or mesangial proliferation (MP) on light microscopy and effacement of foot processes on electron microscopy. Actually the podocyte has become the favourite candidate for constituting the main part of the glomerular filtration barrier. Most cases are steroid sensitive (SSINS). Fifty percents of the latter recur frequently and necessitate a prevention of relapses by nonsteroid drugs. On the contrary to SSINS, steroid resistant nephrotic syndrome (SRINS) leads often to end-stage renal failure. Thirty to forty percents of the latter are associated with mutations of genes coding for podocyte proteins. The rest is due to one or several different circulating factors. New strategies are in development to antagonize the effect of the latter.

## 1. Introduction

Nephrotic syndrome (NS) is an illness consisting in leakage of proteins in urine, resulting in life threatening conditions due hypovolemia, hypercoagulation, and infection. The annual incidence of NS in children in the USA and in Europe has been estimated to be 1–7 per 100,000 children, with a cumulative prevalence of 16 per 100,000 children [1–3]. Nephrotic syndrome in children can be classified according to 3 three groups [3]: secondary, congenital and infantile, and idiopathic. 

Secondary nephrotic syndrome is defined as nephrotic syndrome associated with well-defined diseases that are inflammatory (e.g., lupus nephritis, acute postinfectious glomerulonephritis, IgA nephropathy, Henoch-Schönlein purpura, etc.) or not (e.g., Alport syndrome, focal sclerosis due to reduced nephronic mass resulting from renal scarring, etc.).

Congenital and infantile NSs are occurring before the age of one year and are mostly associated with infections (e.g., syphilis, toxoplasmosis, etc.) or with mutations of genes coding for podocytes proteins and are steroid resistant.

Idiopathic nephrotic syndrome (INS) is the most frequent form of NS in children representing more than 90 percent of cases between 1 and 10 years of age and 50 percent after 10 years of age [1]. INS is defined by the association of the clinical features of NS with renal biopsy findings of diffuse foot process effacement on electron microscopy and minimal changes (called minimal change disease (MCD)), focal segmental glomerulosclerosis (FSGS), or diffuse mesangial proliferation (DMP) on light microscopy [4]. Most patients have histologic findings of MCD. The vast majority of patients with MCD (>90 percent) respond to glucocorticoid therapy whereas only 50 percent of those with DMP and 30 percent of those with FSGS are expected to do so [5]. Clinical findings at presentation differentiate children with MCD from those with other glomerular pathology [1]. The latter include: age younger than six years of age, absence of hypertension, absence of hematuria, normal complement levels, and normal renal function. However, onset of nephrotic syndrome in the first year of life, particularly in the first three months of life, is more likely to be due to a gene mutation and to be resistant to glucocorticoids [6]. 

It is therefore actually generally admitted that a course of glucocorticoids should be given without previous kidney biopsy when the illness has started after the age of one year whereas the upper age limit to do so is generally considered to be 10 years since only 10 percent of patients under 10 years old are steroid resistant in comparison with 20% for the totality of patients less than 18 [4]. 

## 2. The Slit Diaphragm

The INS pathophysiology has been attributed in the past mainly to structural abnormalities and a loss of anionic charges of the glomerular basal membrane (GBM) leading to proteinuria. Actually the podocyte has become the favourite candidate for constituting the main part of the glomerular filtration barrier. The latter is highly specialized, terminally differentiated cells with cytoplasmic extensions, the so-called foot processes anchored on the GBM, forming the slit diaphragm (SD) which is essential in retaining proteins inside the lumen of capillary loop. 

Genetic studies of hereditary forms of NS have led to the identification of proteins playing a crucial role in slit-diaphragm signalling, regulation of actin cytoskeleton dynamics, maintenance of podocyte integrity, and cell-matrix interactions. The latter have been recently reviewed [7]. Structural elements of the SD (nephrin, podocin, and CD2AP) and actin cytoskeleton (a-actinin-4) control podocyte differentiation and survival, cell polarity, and cytoskeletal dynamics. Podocyte and glomerular development are critically regulated by the transcription factor *WT1* and phospholipase C 11 (PLC11) mediated signals. The calcium channel TRPC6, which localizes in membrane lipids supercomplex along podocin, regulates mechanosensation sensed at the SD, whereas the structural component of the GBM, laminin-b2, is essential for podocyte cell-matrix interactions. Podocyte integrity may also be affected by derangements in proteins involved in varied subcellular processes including the mitochondrial respiratory chain, DNA restructuring and repair, and lysosomal function. Finally, identification of novel genetic determinants in glomerular disease, such as high-risk haplotypes in the *MYH9* gene, may also explain the increased risk of some adult patients to glomerular injury.

## 3. Mutations of Genes Coding for Podocytes Proteins

INS secondary to mutations of genes coding for podocytes proteins is typically steroid resistant.

Excellent reviews on this topic [6–9] including the following systematic approach for genetic testing by the group of Antignac [8] have been recently published. A mutation of *NPHS1*, encoding nephrin and responsible for the Finnish-type congenital nephrotic syndrome, is found in most patients presenting with a nephrotic syndrome in the first 3 months of life. The second most frequent mutation in that age group concerns *NPHS2*, which encodes podocin and is particularly frequent in Central Europe. Finally some patients less than 3 months of age present with a mutation of the *WT1* (also often associated with the Denys-Drash and the Frasier syndromes characterized by gonads and genital abnormalities) and/or the PLCE1 genes, particularly when the renal biopsy shows diffuse mesangial sclerosis. In the group of patients starting the illness between 4 and 12 months (infantile nephrotic syndrome) and later on (childhood nephrotic syndrome), *NPHS2* followed by *NPHS1* is the first genes to be tested in nonsyndromic patients presenting SRNS associated with minimal glomerular changes/FSGS in the infantile or childhood period. In the remaining patients with the same histological lesions, genetic testing for *WT1* mutations (exons 8 and 9 in phenotypically female patients) should be performed, while screening for PLCE1 mutations may be considered in some cases (mainly in familial cases). Mutations in the *CD2AP*,* ACTN4*,* TRPC6*, and *INF2 *have also been found anecdotally in childhood SRNS ([10] and for review, [7–9]).

Steroid resitant nephrotic syndrome accompanying several rare syndromes is also reported. A mutation of LAMB2, which encodes laminin beta 2 (a component of the glomerular basement membrane), is responsible for Pierson syndrome. Other rare syndromal conditions are mitochondrial disorders (gene *NA* coding for nonprotein tRNA), Nail-patella syndrome (gene *LMX1B* coding for LIM homeobox transcription factor 1 beta), Schimke immunoosseous dysplasia (*SMARCAL1* coding for SWI/SNF2-related, matrix-associated, actin-dependent regulator of chromatin, subfamily a-like 1), Mandibuloacral dysplasia (*ZMPSTE24* gene coding for the zinc metalloproteinase STE 24), and Galloway-Mowat syndrome (gene *GMSI* coding for *GMSI*). 

Genetic studies are indicated when steroid resistance has been demonstrated in order to withdraw immunosuppression if a mutation is present or in case of congenital nephrotic syndrome before starting any treatment since the great majority of cases is steroid resistant and since steroid treatment can be complicated by severe infectious in neonates [3]. 

The causes of NS recurrence after transplantation in FSGS cases (30–50%) have been recently reviewed and analyzed by the group of Antignac [8]. In contrast to patients with an immune form of NS, those with an inherited structural defect of the glomerular filtration barrier represent a subset of patients for whom the primary disease cannot a priori recur. Surprisingly, recurrence of proteinuria post transplantation has been reported in some patients bearing mutations in the *NPHS1*, *NPHS2*, *ACTN4*, and *WT1* genes (for a review, [8]), and the mechanism of recurrence remains unsolved for a significant proportion of these cases. The most often provided explanation for recurrence of NS is the development of antibodies against the “neoantigen” which is suggested by the fact that treatment with steroids, cyclophosphamide, and plasmapheresis may lead to remission; however, the percentage of graft loss remains significant (for a review, [8]). However, this eventuality is not frequent and genetic studies remain useful before transplantation to precise the risk of posttransplant recurrence and to help for the decision of living kidney donor or not.

## 4. Role of Immunity

INS that is not associated with a mutation of genes coding for podocyte's proteins is actually thought to be the consequence of an immunological dysfunction leading to a circulating factor that modifies the permeability of the glomerular filtration barrier. 

In 1974, Shalhoub [11] proposed that MCNS was a disorder of lymphocyte function with increased plasma levels of a lymphocyte-derived permeability factor. This hypothesis was based on several clinical observations that suggested the involvement of the immune system in the pathogenesis of idiopathic NS, for example, the response to immunosuppressive drugs and the association with Hodgkin disease and with allergy.

The possible role of allergy has been reviewed by van den Berg and Weening [12]. Many reports have been published on patients who developed NS after having experienced allergic reactions to inhaled allergens, with vaccinations, food, and insect stings. Furthermore, the incidence of atopy was reportedly higher in patients with idiopathic NS than in healthy subjects, ranging from 17 to 40% in MCNS patients compared with 10–23% in age-matched control subjects. Allergy is associated with an elevated production of IgE by B-lymphocytes, and several investigators have reported an elevation of IgE in the serum of NS patients. 

The role of circulating factor is particularly suggested by the following observations (for a review, [13]): (1) lmmediate recurrence of proteinuria after transplantation, (2) transfer of proteinuria to fetus, (3) efficacy of plasmapheresis and immunoadsorption in reducing proteinuria, (4) transfer of proteinuria to rat after injection of serum or plasma.

### 4.1. Role of T Lymphocytes and Cytokines

Valuable studies are difficult to obtain since homogeneous patients groups are necessary and it is often not the case since the duration of proteinuria varies at the time of presentation and some patients may have already started treatment. Hyperlipidaemia which is a common complication may activate the immune system, as shown by Lenarsky et al. [14].

Van de Berg and Weening [12] have recently reviewed the immunological role of T cells and cytokines in INS. Frank et al. [15] showed that CD8-positive T-cells of idiopathic NS patients are clonally expanded, which was not observed in healthy controls. Zhang et al. [16] showed high levels of NF-**κ**B (nuclear factor **κ**B) DNA-binding activity in T-cells from untreated MCNS patients during relapse compared with the MCNS patients in remission while treated with immunosuppressants. This points to activation of the T-cells in MCNS. Kimata et al. [17] studied the unstimulated production of cytokines by T-lymphocytes of MCNS patients and found an increased production of IL-13, whereas production of IL-4 was normal. An elevated expression of IL-13 mRNA was shown by Yap et al. [18] using a semiquantitative RT- (reverse transcriptase-) PCR technique. Using a subtractive cDNA library screening technique, Zhang et al. [16] reported differential expression of transcripts involved in the T-cell receptor-mediated complex signaling cascade and a decreased expression of IL-12 receptor 2 mRNA by PBMC in untreated MCNS patients during relapse compared with MCNS patients in remission. The latter studies reveal the involvement of T-cells in the pathogenesis of idiopathic NS and, more specifically, Th2-mediated immunity. Van de Berg and Weening [12] have studied, by quantitative real-time PCR, the expression of IL-1**β**, IL-1ra (IL-1 receptor antagonist), IL-2, IL-4, IL-5, IL-9, IL-10, IL-13, TNF-**α**, and IFN-**γ**by PBMC from patients with MCNS during relapse and remission and from a control group of patients with NS primarily caused by endogenous alterations within the glomerular filter, for instance, mutations in the genes encoding nephrin and podocin. Out of the cytokines studied, only the expression of IL-10 and IL-13 mRNA was significantly upregulated in relapsing MCNS patients when compared with MCNS patients in remission. The latter authors and others (for a review, [12]) have shown that podocytes constitutively express functional transmembrane receptor complexes for IL-4, IL-10, IL-13, and TNF-**α**. The possible role of IL-13 is also suggested by the NS rat model of Lai et al. [19]. IL-13 was overexpressed in Wistar rats through transfection of a mammalian expression vector cloned with the rat IL-13 gene, into the quadriceps by in vivo electroporation. The IL-13-transfected rats showed significant albuminuria, hypoalbuminemia, and hypercholesterolemia when compared with control rats. No significant histologic changes were seen in glomeruli of IL-13-transfected rats. However, electron microscopy showed up to 80% of podocyte foot process fusion. Glomerular gene expression was significantly downregulated for nephrin, podocin, CD 80, and dystroglycan. Immunofluorescence staining intensity was reduced for nephrin, podocin, and dystroglycan IL-4R alpha in IL-13-transfected rats compared with controls. 

Abdel-Hafez et al. [20] suggest in review of the literature and of their own results that the relation between allergy and INS could be the stimulation by IL-13 of the expression of CD 80 on podocytes. They report that urinary CD80 levels are increased in patients with MCD during relapse and return to normal after remission. They also have preliminary evidence that the source of the CD80 is the podocyte because they found, by using immunohistochemical staining, that CD80 was expressed by podocytes in kidney biopsy specimens from patients with MCD in relapse. 

The successful treatment of SRNS on native kidney or after kidney transplantation with anti-TNF*α* antibodies strongly suggests that this cytokine participates to the pathogenesis of some types of idiopathic nephrotic syndrome [21, 22]. It is also suggested by high levels of TNF*α* in patients with active disease and TNF*α* normalization with remission and by an animal model of NS that is controled by anti-TNF*α* agents (for a review, [21, 22]).

### 4.2. Role of B Lymphocytes

The beneficial treatment by rituximab, a monoclonal antibody directed against CD20, in difficult SSINSs suggest, a role for B cells in INS [23–25]. Sellier-Leclerc et al [25] underline that most arguments gathered by Shalhoub [11] in support of T cell dysfunction have a counterpart to supporting B cell dysfunction and that the contribution of B cells and the potential role of immunoglobulin chains in modifying the glomerular permeability to protein in children with steroid-sensitive nephrotic syndrome have been repeatedly reported [25]. In addition, B cells may be involved through an unidentified antibody-independent pathway, that might be a control on T cells [26]. 

## 5. Possible Permeability Factors

Aside cytokines other factors have been suspected to be involved in the pathogenesis of INS. One of them consists in radical oxygen species (ROS) that have been largely studied by the group of Ghiggeri (for a review, [27]). Some experimental models of nephrotic syndrome result from substances as puromycine and adriamycin that induce oxidative stress in glomeruli. Furthermore the injection of H_2_O_2_ induces proteinuria in rats and NO prevents the increase of permeability to albumin induced by the production TNF alpha-induced O_2_- production in an isolated rat glomeruli system [28]. Active focal segmental glomerulosclerosis is associated with massive oxidation of plasma albumin [27]. Recently, Bertelli et al. [29] demonstrated a 10-fold increase of ROS production by resting PMN in INS compared to normal PMN. When PMNs were separated from other cells, ROS increased significantly in all conditions while a near normal production was restored by adding autologous cells and/or supernatants in controls, vasculitis, and postinfectious glomerulonephritis but not in INS. The second finding was that the oxidative burst by PMN was regulated highly by T lymphocytes, mainly Tregs, by means of soluble factors and that this regulatory circuit was altered in INS.

The group of Moin Saleem has intensively studied the possible pathophysiological role of hemopexin (Hx) in INS [30, 31]. Hx is well described as a heme-scavenging protein. It is predominantly produced in the liver, and it increases in the acute phase reaction to inflammation or infection. Plasma-purified and recombinant Hx has been shown to have serine protease activity. It has been suggested that in normal conditions circulating Hx is inactive but under certain circumstances Hx becomes activated as a serine protease. Activated Hx has been shown to have dramatic effects on the glomerular filtration barrier. Kidney sections incubated with Hx have a reduction of the anionic layer and reduced sialoglycoproteins. In vivo, activated Hx induced reversible proteinuria in rats parallel to podocyte foot process effacement. Activated hemopexin is increased in children with minimal change nephrotic syndrome [31]. In vitro within 30 minutes of treatment with hemopexin, actin reorganized from stress fibers to cytoplasmic aggregates and membrane ruffles in wild-type podocytes [30, 31]. This process is nephrin dependent since it did not occur in nephrin-deficient podocytes and in cells that do not express nephrin and was inhibited by preincubation with human plasma. In addition, hemopexin led to a selective increase in the passage of albumin across monolayers of glomerular endothelial cells and to a reduction in glycocalyx. What remains to be elucidated is the primary events leading to the activation of Hx. A possibility resides in the inhibition of Hx inhibitors or in their leakage in urine. In the latter case, Hx activation should be only a secondary event depending on the increased permeability of the glomerular filtration barrier to proteins. 

The group of Virginia Savin in US has studied and characterized the circulating factor in FSGS by analyzing the plasma of patients presenting with a posttransplant relapse (for a review, [13]). Those studies are based on standard methods of biochemical purification and analyses of molecular characteristics followed by gel electrophoresis and mass spectrometry. They have used a functional assay of permeability activity with isolated rat glomeruli that shows changes in the glomerular capillary permeability to albumin after incubation with the patient plasma or serum. This assay has made it possible to perform sequential purification steps and select fraction(s) with enhanced activity. Using galactose as an effective affinity material to enrich activity of FSGS plasma they reported that cardiotrophin-like cytokine factor 1 (CLC-1; encoded by *CLCF1*), a member of the interleukin 6 family, is present in the enriched fraction of FSGS plasma, and that CLC-1 increases glomerular P_alb_, and its injection causes proteinuria in rats. 

## 6. Treatment

Pathophysiological consequences of nephrotic syndrome as hypovolemia, acute renal failure, edema, hypercoagulation, and infections should be treated symptomatically. 

The basis of treatment of INS by steroids was the early hypothesis of the implication of an immunological factor in the pathophysiology of the disease. The latter has also lead to use immunosuppressive drugs to prevent relapses. However, recent experiments using podocytes in vitro have shown that steroids and cyclosporine, aside their effect on the immunological system, may also act directly on the podocyte to stabilize its structure [32, 33]. 

Recovery of a normal permeability to proteins of the glomerular filtration barrier is rendered possible by steroids in the majority of cases. The Cochrane reviews that use severe criteria of quality to analyze results of clinical studies are very useful for the clinician. 

### 6.1. Steroid Sensitive Idiopathic Nephrotic Syndrome

The treatment of the primary immunological cause of INS resides in the use of steroids. The latter allows the differentiation between steroid sensitive and steroid resistant nephrotic syndrome. Different protocols are used according to countries. Initial dosage of 60 mg/m^2^/d prednisone or prednisolone pursued by alternate day administration to reduce side effects is a common feature. Steroids protocols are mostly differing by duration, way of tapering, and definition of steroid resistance. A 2007 Cochrane review [34] has analyzed the results of the literature concerning the best initial steroid treatment. The authors conclude that in their first episode of SSNS patients should be treated for at least three months with an increase in benefit for up to seven months of treatment. For a baseline risk for relapse following the first episode of 60% with two months of therapy, daily prednisone or prednisolone given for four weeks followed by alternate-day therapy for six months would reduce the number of children relapsing by 33%. However, it remains to be known if the time of administration or the cumulative steroid dosage is the most important determinant. This question is addressed partly in a double blind RCT comparing the effect of the same cumulative prednisone dosage given during 3 or 5 months by a study actually going on at the University of Rotterdam (The Netherlands). 

In case of frequently relapsing or steroid-dependent nephrotic syndrome, repeated and prolonged high dosage of prednisone might lead to severe side effects as growth retardation, osteoporosis, infections, diabetes, cataract, hypertension, hirsutism, and Cushing aspect. In order to avoid the latter, different nonsteroid treatments have been used. The 2008 Cochrane analysis of the already published results of the latter concludes that eight-week courses of cyclophosphamide and prolonged courses of cyclosporin and of levamisole reduce the risk of relapse in children with relapsing SSNS compared with corticosteroids alone [35]. They conclude also that clinically important differences in efficacy are possible and that further comparative studies are still needed. Furthermore side effects should be taken into account by the clinician. Infection, infertility, and possibly cancer at long term for cyclophosphamide and hypertension, hirsutism, and chronic renal failure for cyclosporine lead the clinicians to consider alternative treatments. A preliminary study comparing cyclosporine to mycophenolate mofetyl suggested that the latter could replace cyclosporine in the majority of cases [36]. Recently, the successful use of rituximab, a monoclonal anti-CD20 antibody, has been reported to prevent relapses in difficult steroid-dependent SSINS in several reports [23–25]. However, the latter should be confirmed in a RCT to better define efficacy, modalities (administration during relapse or after remission induction, used alone or in combination), indications, and safety. Presently, rituximab has to be considered as the last option, for patients who cannot be managed properly despite alternate day steroids, MMF, and anticalcineurins (because of ongoing relapses and/or side effects of drugs and/or noncompliance).

### 6.2. Steroid Resistant Idiopathic Nephrotic Syndrome

The last Cochrane review on this topic [37] reports that when cyclosporin was compared to placebo or no treatment, there was a significant increase in the number of children who achieved complete remission. Cyclosporin also significantly increased the number of children, who achieved complete or partial remission compared with IV cyclophosphamide. There was no improvement with other immunosuppressive agents. However the number of studies was small. More research is needed. The analysis of results of treatment of SRINS is complicated by the heterogeneity of steroid resistance definition according to protocols and countries. Another issue concerns the lack of studies considering the presence of mutations of podocytes proteins to explain different responses to treatment. It seems indeed important to distinguish the latter from SRINS possibly due to a circulating factor since any immunological treatment is most probably ineffective in the former. When no mutation is detected, a circulating factor might be suspected, particularly when a recurrence is observed after kidney transplantation. In that case, removal of the latter by plasma exchange can be effective but recurrence is often observed when treatment is interrupted. The future treatment strategy will imply the determination of the latter factor and the use of specific treatment to antagonize its effect. Based on this principle, RCTs studying the effect of galactose and anti-TNF Ab have already been initiated by the FONT study group [38]. Another approach used by the latter group is to antagonize the effect of TGF*β*, a cytokine playing a major role in fibrotic processes [39].

## References

[1] (1978). Nephrotic syndrome in children: prediction of histopathology from clinical and laboratory characteristics at time of diagnosis. A report of the International Study of Kidney Disease in Children. *Kidney International*.

[2] McKinney PA, Feltbower RG, Brocklebank JT, Fitzpatrick MM (2001). Time trends and ethnic patterns of childhood nephrotic syndrome in Yorkshire, UK. *Pediatric Nephrology*.

[3] Niaudet P, Avner ED, Harmon WE, Neasden P ( 2004). Steroid-sensitive nephrotic syndrome in children. *Paediatric Nephrology*.

[4] White RH, Glasgow EF, Mills RJ (1970). Clinicopathological study of nephrotic syndrome in childhood. *The Lancet*.

[5] Barnett HL, Edelmann CM, Greifer I (1981). The primary nephrotic syndrome in children. Identification of patients with minimal change nephrotic syndrome from initial response to prednisone. A report of the international study of kidney disease in children. *Journal of Pediatrics*.

[6] Hinkes BG, Mucha B, Vlangos CN (2007). Nephrotic syndrome in the first year of life: two thirds of cases are caused by mutations in 4 genes (NPHS1, NPHS2, WT1, and LAMB2). *Pediatrics*.

[7] Machuca E, Benoit G, Antignac C (2009). Genetics of nephrotic syndrome: connecting molecular genetics to podocyte physiology. *Human Molecular Genetics*.

[8] Benoit G, MacHuca E, Antignac C (2010). Hereditary nephrotic syndrome: a systematic approach for genetic testing and a review of associated podocyte gene mutations. *Pediatric Nephrology*.

[9] Löwik MM, Groenen PJ, Levtchenko EN, Monnens LA, Van Den Heuvel LP (2009). Molecular genetic analysis of podocyte genes in focal segmental glomerulosclerosis-a review. *European Journal of Pediatrics*.

[10] Heeringa SF, Möller CC, Du J (2009). A novel TRPC6 mutation that causes childhood FSGS. *PLoS ONE*.

[11] Shalhoub RJ (1974). Pathogenesis of lipoid nephrosis: a disorder of T cell function. *The Lancet*.

[12] van den Berg JG, Weening JJ (2004). Role of the immune system in the pathogenesis of idiopathic nephrotic syndrome. *Clinical Science*.

[13] McCarthy ET, Sharma M, Savin VJ (2010). Circulating permeability factors in idiopathic nephrotic syndrome and focal segmental glomerulosclerosis. *Clinical Journal of the American Society of Nephrology*.

[14] Lenarsky C, Jordan SC, Ladisch S (1982). Plasma inhibition of lymphocyte proliferation in nephrotic syndrome: correlation with hyperlipidemia. *Journal of Clinical Immunology*.

[15] Frank C, Herrmann M, Fernandez S (2000). Dominant T cells in idiopathic nephrotic syndrome of childhood. *Kidney International*.

[16] Zhang SY, Audard V, Fan Q, Pawlak A, Lang P, Sahali D (2011). Immunopathogenesis of idiopathic nephrotic syndrome. *Contributions to Nephrology*.

[17] Kimata H, Fujimoto M, Furusho K (1995). Involvement of interleukin (IL)-13, but not IL-4, in spontaneous IgE and IgG4 production in nephrotic syndrome. *European Journal of Immunology*.

[18] Yap HK, Cheung W, Murugasu B, Sim SK, Seah CC, Jordan SC (1999). Th1 and Th2 cytokine mRNA profiles in childhood nephrotic syndrome: evidence for increased IL-13 mRNA expression in relapse. *Journal of the American Society of Nephrology*.

[19] Lai KW, Wei CL, Tan LK (2007). Overexpression of interleukin-13 induces minimal-change-like nephropathy in rats. *Journal of the American Society of Nephrology*.

[20] Abdel-Hafez M, Shimada M, Lee PY, Johnson RJ, Garin EH (2009). Idiopathic nephrotic syndrome and atopy: is there a common link?. *American Journal of Kidney Diseases*.

[21] Leroy S, Guigonis V, Bruckner D (2009). Successful anti-TNF*α* treatment in a child with posttransplant recurrent focal segmental glomerulosclerosis. *American Journal of Transplantation*.

[22] Raveh D, Shemesh O, Ashkenazi YJ, Winkler R, Barak V (2004). Tumor necrosis factor-*α* blocking agent as a treatment for nephrotic syndrome. *Pediatric Nephrology*.

[23] Guigonis V, Dallocchio A, Baudouin V (2008). Rituximab treatment for severe steroid- or cyclosporine-dependent nephrotic syndrome: a multicentric series of 22 cases. *Pediatric Nephrology*.

[24] Gulati A, Sinha A, Jordan SC (2010). Efficacy and safety of treatment with rituximab for difficult steroid-resistant and -dependent nephrotic syndrome: multicentric report. *Clinical Journal of the American Society of Nephrology*.

[25] Sellier-Leclerc AL, MacHer MA, Loirat C (2010). Rituximab efficiency in children with steroid-dependent nephrotic syndrome. *Pediatric Nephrology*.

[26] Deola S, Panelli MC, Maric D (2008). Helper B cells promote cytotoxic T cell survival and proliferation independently of antigen presentation through CD27/CD70 interactions. *Journal of Immunology*.

[27] Musante L, Candiano G, Petretto A (2007). Active focal segmental glomerulosclerosis is associated with massive oxidation of plasma albumin. *Journal of the American Society of Nephrology*.

[28] Sharma M, McCarthy ET, Savin VJ, Lianos EA (2005). Nitric oxide preserves the glomerular protein permeability barrier by antagonizing superoxide. *Kidney International*.

[29] Bertelli R, Trivelli A, Magnasco A (2010). Failure of regulation results in an amplified oxidation burst by neutrophils in children with primary nephrotic syndrome. *Clinical and Experimental Immunology*.

[30] Coward RJM, Foster RR, Patton D (2005). Nephrotic plasma alters slit diaphragm-dependent signaling and translocates nephrin, podocin, and CD2 associated protein in cultured human podocytes. *Journal of the American Society of Nephrology*.

[31] Bakker WW, van Dael CML, Pierik LJWM (2005). Altered activity of plasma hemopexin in patients with minimal change disease in relapse. *Pediatric Nephrology*.

[32] Faul C, Donnelly M, Merscher-Gomez S (2008). The actin cytoskeleton of kidney podocytes is a direct target of the antiproteinuric effect of cyclosporine A. *Nature Medicine*.

[33] Xing CY, Saleem MA, Coward RJ, Ni L, Witherden IR, Mathieson PW (2006). Direct effects of dexamethasone on human podocytes. *Kidney International*.

[34] Hodson EM, Willis NS, Craig JC (2007). Corticosteroid therapy for nephrotic syndrome in children. *Cochrane Database of Systematic Reviews*.

[35] Hodson EM, Willis NS, Craig JC (2008). Non-corticosteroid treatment for nephrotic syndrome in children. *Cochrane Database of Systematic Reviews*.

[36] Dorresteijn EM, Kist-van Holthe JE, Levtchenko EN, Nauta J, Hop WCJ, van der Heijden AJ (2008). Mycophenolate mofetil versus cyclosporine for remission maintenance in nephrotic syndrome. *Pediatric Nephrology*.

[37] Hodson EM, Willis NS, Craig JC (2010). Interventions for idiopathic steroid-resistant nephrotic syndrome in children. *Cochrane Database of Systematic Reviews*.

[38] Trachtman H, Vento S, Gipson D (2011). Novel therapies for resistant focal segmental glomerulosclerosis (FONT) phase II clinical trial: study design. *BMC Nephrology*.

[39] Trachtman H, Fervenza FC, Gipson DS (2011). A phase 1, single-dose study of fresolimumab, an anti-TGF-*β* antibody, in treatment-resistant primary focal segmental glomerulosclerosis. *Kidney International*.

